# Synthesis, crystal structure and properties of chlorido­tetra­kis­(pyridine-3-carbo­nitrile)­thio­cyanato­iron(II)

**DOI:** 10.1107/S205698902300988X

**Published:** 2023-11-21

**Authors:** Asmus Müller-Meinhard, Inke Jess, Christian Näther

**Affiliations:** a Institut für Anorganische Chemie, Universität Kiel, Max-Eyth. Str. 2, 24118 Kiel, Germany; Institute of Chemistry, Chinese Academy of Sciences

**Keywords:** synthesis, crystal structure, mixed iron thio­cyanate chloride complex, powder diffraction, thermal properties

## Abstract

Reaction of FeCl_2_·4H_2_O with KSCN and 3-cyano­pyridine (pyridine-3-carbo­nitrile) in ethanol accidentally leads to the formation of single crystals of Fe(NCS)(Cl)(3-cyano­pyridine)_4_ or [FeCl(NCS)(C_6_H_4_N_2_)_4_]. The asymmetric unit of this compound consists of one Fe^II^ cation, one chloride and one thio­cyanate anion that are located on a fourfold rotation axis as well as of one 3-cyano­pyridine coligand in a general position. The Fe^II^ cations are sixfold coordinated by one chloride anion and one terminally N-bonding thio­cyanate anion in *trans*-positions and four 3-cyano­pyridine coligands that coordinate *via* the pyridine N atom to the Fe^II^ cations. The complexes are arranged in columns with the chloride anions, with the thio­cyanate anions always oriented in the same direction, which shows the non-centrosymmetry of this structure. No pronounced inter­molecular inter­actions are observed between the complexes. Initially, FeCl_2_ and KSCN were reacted in a 1:2 ratio, which lead to a sample that contains the title compound as the major phase together with a small amount of an unknown crystalline phase, as proven by powder X-ray diffraction (PXRD). If FeCl_2_ and KSCN is reacted in a 1:1 ratio, the title compound is obtained as a nearly pure phase. IR investigations reveal that the CN stretching vibration for the thio­cyanate anion is observed at 2074 cm^−1^, and that of the cyano group at 2238 cm^−1^, which also proves that the anionic ligands are only terminally bonded and that the cyano group is not involved in the metal coordination. Measurements with thermogravimetry and differential thermoanalysis reveal that the title compound decomposes at 169°C when heated at a rate of 4°C min^−1^ and that the 3-cyano­pyridine ligands are emitted in two separate poorly resolved steps. After the first step, an inter­mediate compound with the composition Fe(NCS)(Cl)(3-cyano­pyridine)_2_ of unknown structure is formed, for which the CN stretching vibration of the thio­cyanate anion is observed at 2025 cm^−1^, whereas the CN stretching vibration of the cyano group remain constant. This strongly indicates that the Fe^II^ cations are linked by μ-1,3-bridg­ing thio­cyanate anions into chains or layers.

## Abstract 2

In the crystal structure of the title compound, Fe(NCS)(Cl)(3-cyano­pyridine)_4_ (3-cyano­pyridine = pyridine-3-carbo­nitrile) the Fe^II^ cations are octa­hedrally coordinated by one terminal N-bonding thio­cyanate anion, one chloride anion and four 3-cyano­pyridine coligands that coordinate with the pyridine N atom to the iron centers. Upon heating the 3-cyano­pyridine coligands are emitted in two separate steps, leading to the formation of an inter­mediate compound with the composition Fe(NCS)(Cl)(3-cyano­pyridine)_2_.

## 1. Chemical context

Thio­cyanate anions are versatile ligands, which show a number of different coordination modes, leading to a pronounced structural variability. This ligand can act as a monocoordinating ligand, which in most cases leads to the formation of complexes that are of inter­est, for example in the field of spin-crossover compounds, which is especially the case with Fe(NCS)_2_ (Gütlich *et al.*, 2000 ▸; Naggert *et al.*, 2015 ▸; Senthil Kumar & Ruben Kuppusamy, 2017 ▸; Hogue *et al.*, 2018 ▸). Moreover, this anionic ligand is able to mediate magnetic exchange and therefore, compounds with bridging thio­cyanate anions are also of inter­est (Palion-Gazda *et al.*, 2015 ▸; Mekuimemba *et al.*, 2018 ▸). In this context, compounds based on Co(NCS)_2_ are of special importance because of the large magnetic anisotropy of Co^II^ (Mautner *et al.*, 2018 ▸; Wöhlert *et al.*, 2013 ▸; Rams *et al.*, 2020 ▸). All these are reasons why the inter­est in the synthesis, structures and properties of thio­cyanate coordination compounds is still very high. In our own investigations, we are especially inter­ested in coordination compounds with Mn^II^, Fe^II^, Co^II^ and Ni^II^ cations.

The synthesis of such thio­cyanate coordination compounds with manganese, cobalt and nickel is usually very easy because their thio­cyanate salts are commercially available or can easily be prepared and stored for a long time, which is not the case for Fe(NCS)_2_. For the synthesis of coordination compounds with this cation, Fe(NCS)_2_ is usually prepared *in situ*, for example by the reaction of an Fe^II^ salt such as FeCl_2_ or FeSO_4_ with KSCN, which afterwards reacts with the organic ligand to form the desired thio­cyanate compound. The potassium salt formed in this reaction can finally be removed, for example by washing the residue with water. We have used this procedure many times for the preparation of new Fe(NCS)_2_ compounds, and it usually leads to pure samples (Wöhlert *et al.*, 2013 ▸; Werner *et al.*, 2015*a* ▸,*b* ▸).

However, in the course of our systematic investigations we became inter­ested in the synthesis of Fe(NCS)_2_ precursor complexes with 3-cyano­pyridine as coligand, for which corresponding compounds with Mn^II^ and Ni^II^ had already been investigated by us (Krebs *et al.*, 2021 ▸, 2023 ▸). In this work we investigated whether 3-cyano­pyridine-rich complexes with terminally N-bonded thio­cyanate anions can be prepared and transformed into 3-cyano­pyridine-deficient complexes with bridging thio­cyanate anions by thermal decomposition. For a number of complexes with Ni(NCS)_2_ we found that they transform into a new compound with the composition Ni(NCS)_2_(3-cyano­pyridine)_2_, in which the metal cations are linked by the thio­cyanate anions into layers and in which the 3-cyano­pyridine ligand is only terminally bonded (Krebs *et al.*, 2021 ▸). Surprisingly, corresponding complexes with Mn(NCS)_2_ transform into an unusual compound with the composition {[Mn(NCS)_2_]_3_(3-cyano­pyridine)_4_}_n_, which is isotypic to the corresponding compound with Cd(NCS)_2_ already reported in the literature (Jochim *et al.*, 2020*a* ▸,*b* ▸) and which consists of Mn(NCS)_2_ chains that are connected by some bridging 3-cyano­pyridine ligands into layers, whereas some others are still terminally bonded (Krebs *et al.*, 2023 ▸). The reason for the differences in the thermal behavior is unclear, but the question arises whether cations in between Mn^II^ and Ni^II^ will show a thermal behavior similar to that of Mn^II^ or Ni^II^. We therefore decided to attempt to prepare thio­cyanate complexes based on Fe(NCS)_2_ and 3-cyano­pyridine.

For the synthesis of such compounds we reacted FeCl_2_ and FeSO_4_ with KSCN, which led to the formation of crystalline products that were identified by single-crystal X-ray diffraction. This proves that in the batch obtained from FeCl_2_·6H_2_O, a compound with the composition Fe(NCS)(Cl)(3-cyano­pyridine)_4_ was accidentally obtained, in which both thio­cyanate and chloride anions are present. In contrast, with FeSO_4_, the desired compounds with composition Fe(NCS)_2_(3-cyano­pyridine)_4_ and Fe(NCS)_2_(3-cyano­pyridine)_2_(H_2_O)_2_·2(3-cyano­pyridine) were obtained (Näther *et al.*, 2023 ▸). In this context, it is noted that compounds with transition metals coordinated by a halide anion and a thio­cyanate anion with 3-cyano­pyridine are unknown. In general, only one Fe compound is found in the CSD (see *Database survey*) in which the Fe^II^ cation is coordinated by one chloride anion, one thio­cyanate anion and an N-donor ligand (Horng & Lee, 1999 ▸). Concerning the synthesis of such compounds, most compounds reported in literature were prepared by the reaction of one equivalent of a transition metal–halide salt with one or two equivalents of potassium or ammonium thio­cyanate, very similar to the synthesis of the title compound, but in none of these publications was the purity of the compounds investigated by X-ray powder diffraction (PXRD). 
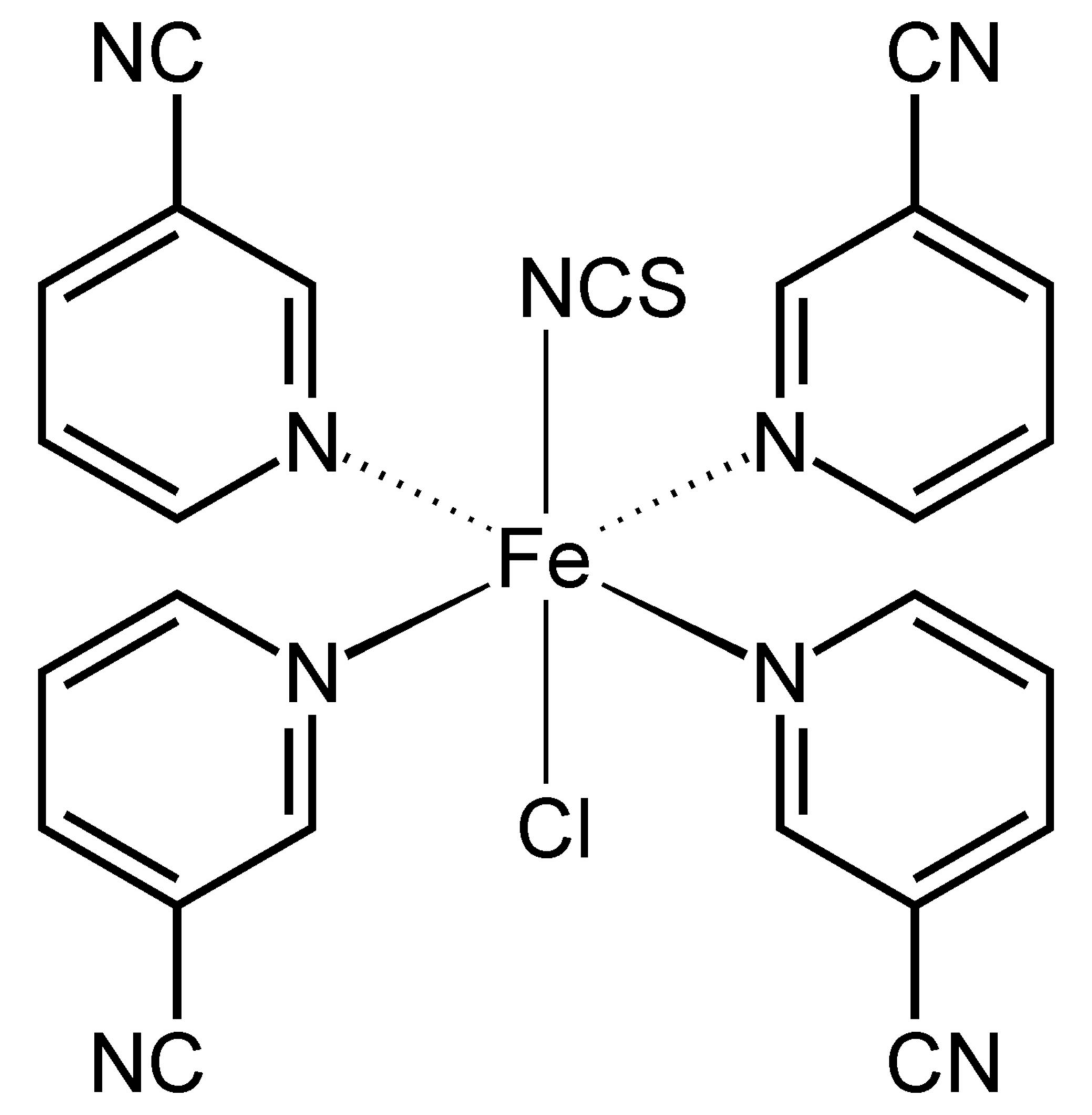


## 2. Structural commentary

**Figure 1:**
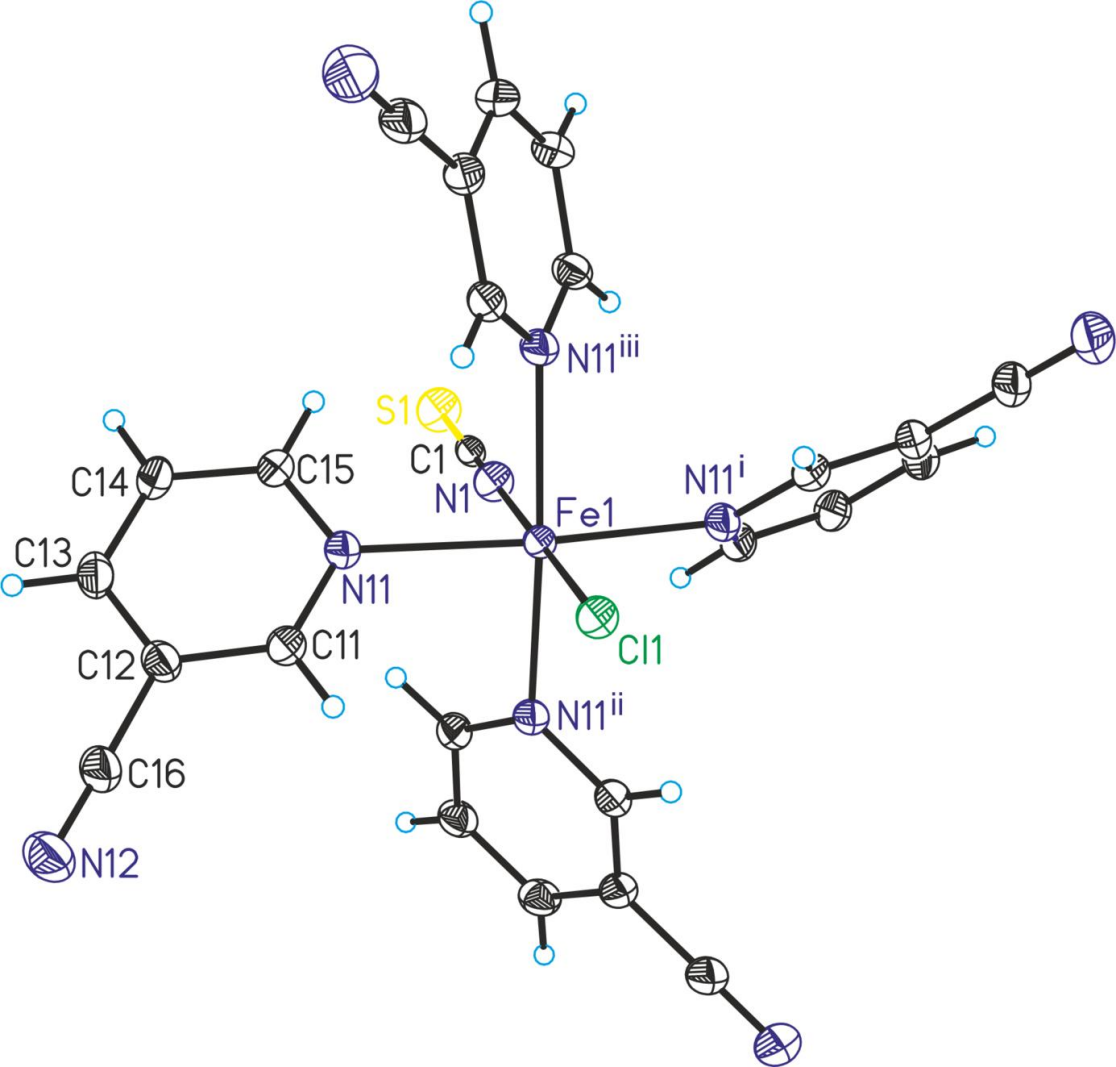
Crystal structure of the title compound with labeling and displacement ellipsoids drawn at the 50% probability level. Symmetry codes for the generation of equivalent atoms: (i) *y*, −*x* + 1, *z*; (ii) −*x* + 1, −*y* + 1, *z*; (iii) −*y* + 1, *x*, *z*.

The asymmetric unit of the title compound, Fe(NCS)(Cl)(3-cyano­pyridine)_4_, consists of one iron cation, one thio­cyanate anion and one chloride anion that are located on a fourfold rotation axis, as well as of one 3-cyano­pyridine coligand that occupies a general position (Fig. 1 ▸). In the crystal structure, the Fe^II^ cations are coordinated by one terminally N-bonded thio­cyanate anion and one chloride anion in *trans*-positions and four symmetry-related 3-cyano­pyridine coligands that are coordinated *via* the pyridine N atom to the Fe centers (Fig. 1 ▸). As a result of symmetry, all four Fe—N bond lengths to the coligands are identical and correspond to literature values. The bonding angles deviate from the ideal values, which is especially the case for the Cl—Fe—N_3-cyano­pyridine_ and the N_NCS_—Fe—N_3-cyano­pyridine_ angle, whereas the N—Fe—N angles of neighboring 3-cyano­pyridine coligands are close to 90° (Table 1 ▸). Therefore, the octa­hedra are slightly distorted. As a result of steric repulsion, the 3-cyano­pyridine ring planes are not coplanar and are rotated by about 70°.

## 3. Supra­molecular features

**Figure 2:**
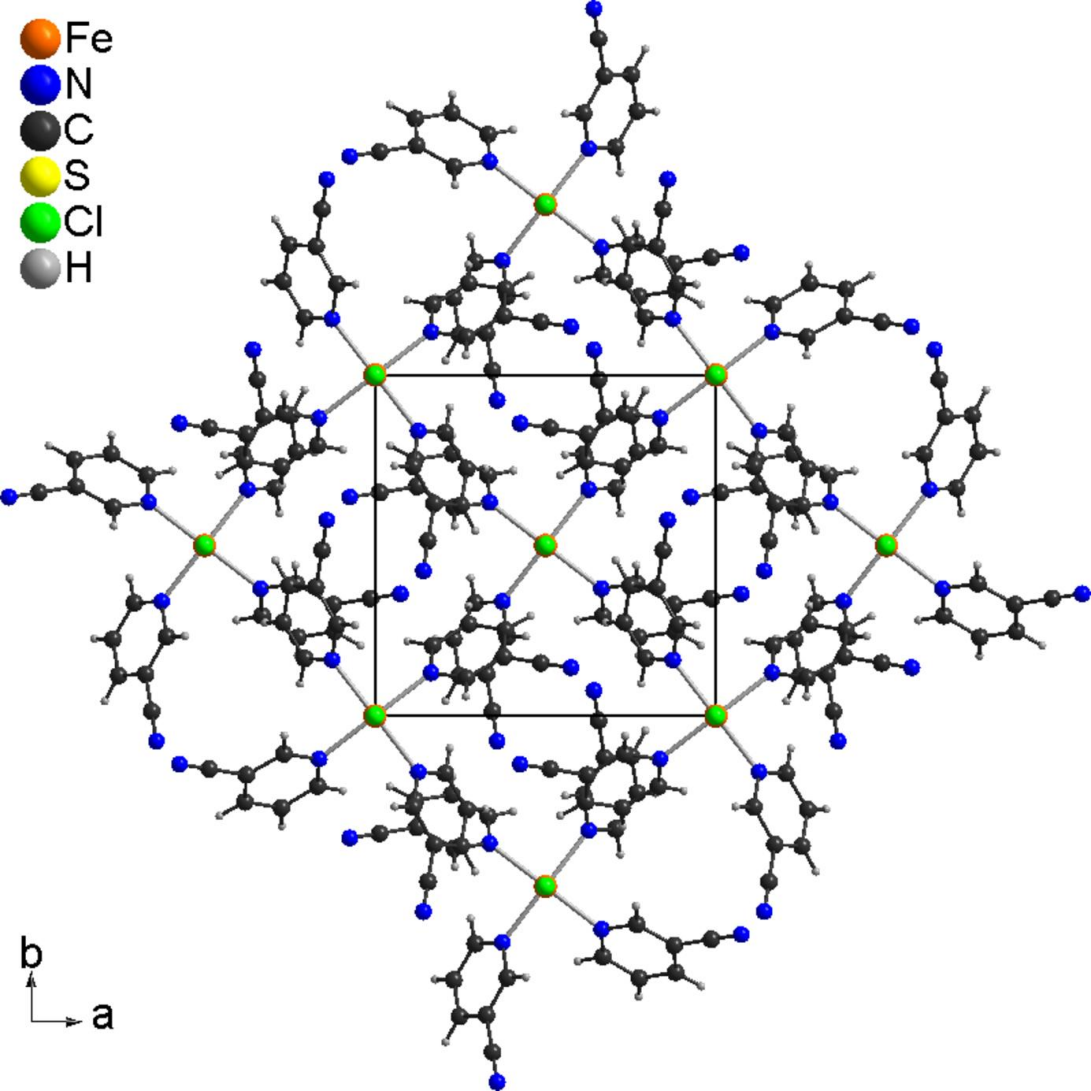
Crystal structure of the title compound in a view along the crystallographic *c*-axis direction.

**Figure 3:**
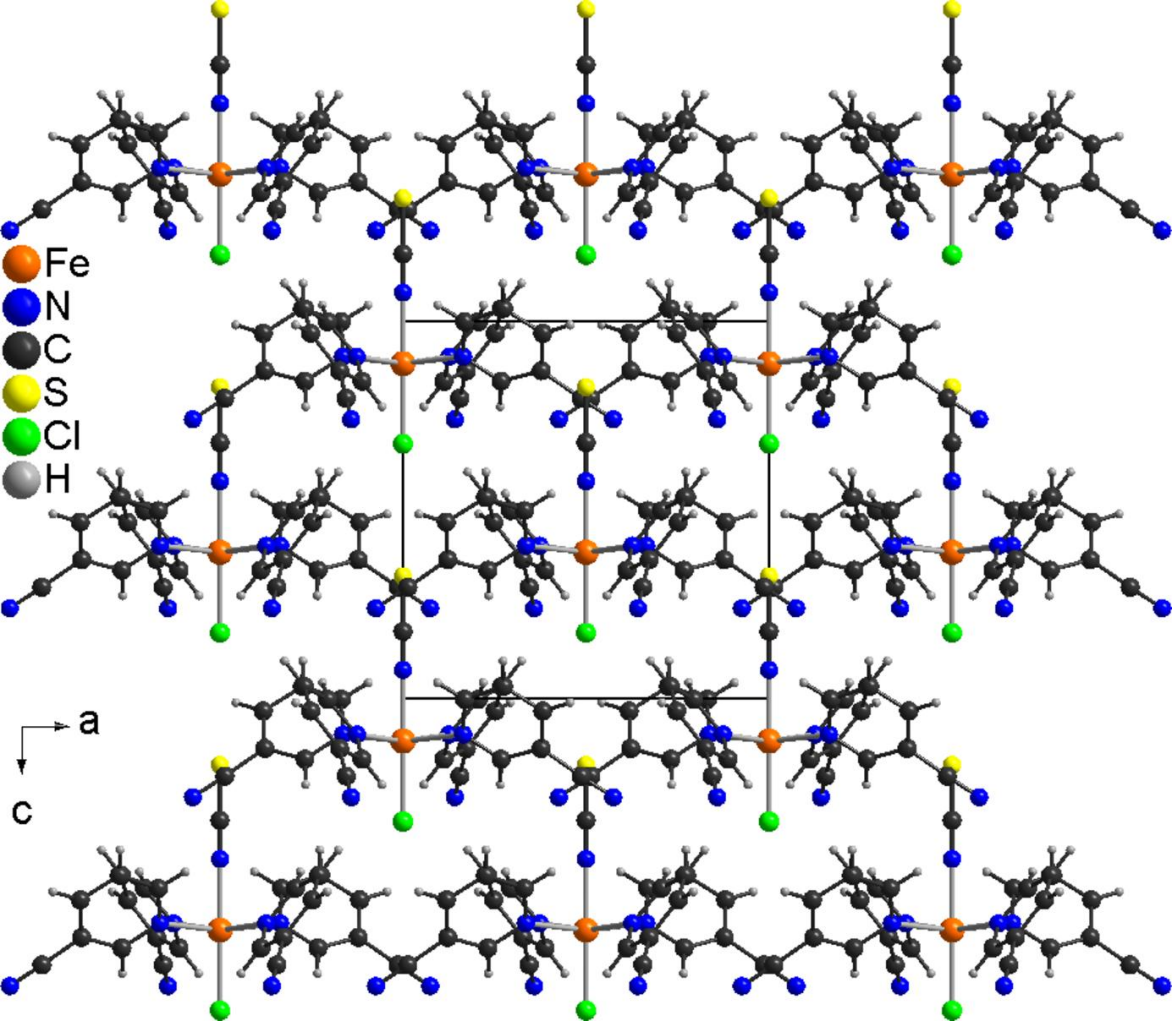
Crystal structure of the title compound in a view along the crystallographic *b*-axis direction, showing the non-centrosymmetry of the structure.

In the crystal, the discrete complexes are arranged in columns that elongate in the *c*-axis direction (Fig. 2 ▸). From a view along the *b*-axis, it is obvious that all chloride anions and thio­cyanate anions always point in the same direction, which proves the non-centrosymmetry of this structure (Fig. 3 ▸). There are no pronounced directional inter­actions between the complexes, except for two C—H⋯N inter­actions but, from the bond lengths and angles, it is obvious that they do not correspond to significant inter­actions (Table 2 ▸).

## 4. Database survey

A search in the Cambridge Structural Database (CSD version 5.43, last update November 2023; Groom *et al.*, 2016 ▸) using ConQuest (Bruno *et al.*, 2002 ▸) revealed that no complexes consisting of a transition-metal cation coordinated by a halide anion, a thio­cyanate anion and a 3-cyano­pyridine ligand are known.

Searching for compounds with iron coordinated by a thio­cyanate and a halide anion, only one structure was found. In (μ_2_-*N*,*N*,*N*′,*N*′-tetra­kis­(2-benzimidazolylmeth­yl)-2-oxy-1,3-di­amino­propane)­dichloro­diiso­thio­cyanato­diiron(iii) chloride tetra­hydrate (refcode: HOJLEX, Horng & Lee, 1999 ▸), the iron cations are octa­hedrally coordinated by one chloride anion and one thio­cyanate anion in *cis*-positions, as well as three N and one O atoms of the organic ligand. Pairs of Fe^II^ cations are linked by a μ-1,1(*O*,*O*)-bridging O atom into dinuclear units.

After expanding the search to compounds in which a transition-metal cation is coordinated by a thio­cyanate anion, a halide anion and a pyridine derivate, some more structures were found, most of them with chloride anions. This includes discrete complexes with the composition *M*(NCS)(*X*)(*L*) (*M* = Cu, Co, Zn, *X* = Cl, Br) in which the metal cation is coordinated by one thio­cyanate anion, one halide anion and one tridentate ligand {*L* = 2,6-bis­(pyridin-2-yl)-3,5-bis­(pyridin-2-yl)pyrazine, refcode: FEPKEU; Al-Assy & Mostafa, 2023 ▸; *L* = 4-meth­oxy-*N*-[(pyridin-2-yl)methyl­idene]benzene-1-carbohydrazonato, refcode: FIRPAA; Yu *et al.*, 2018 ▸; *L* = 2-[1-(pyridin-2-yl)ethyl­idene]hydrazinecarboximidamide, refcode: IQEFER; Vojinović-Ješić *et al.*, 2016 ▸; *L* = 2-amino-*N*′-[(pyridin-2-yl)methyl­idene]benzohydrazide, refcode: KEPPII; Zhang *et al.*, 2022 ▸; *L* = 2,2′-(pyridine-2,6-di­yl)bis-1*H*-benzimidazole, refcode: QEHRAY; Machura *et al.*, 2012 ▸; *L* = *N*,*N*-dimethyl-*N*′-(1-pyridinyl­methyl­idene)propane-1,3-di­amine, refcode: YIJYEW; Sun, 2006 ▸; *L* = *N*-methyl-*N*′-[1-(2-pyrid­yl)ethyl­idene]ethane-1,2-di­amine-κ^3^ *N*,*N*′,*N*′′, refcode: DUR­FOM; Liu, 2010 ▸}.

Additional discrete complexes of the composition *M*(NCS)Cl(*L*)_2_ (*M* = Cu, Co) are found in which the metal cations are octa­hedrally coordinated by one thio­cyanate anion, one chloride anion and two bidentate ligands [*L*= 2-(pyridin-2-yl)-1*H*-benzimidazole, refcode: VEJHAW; Kumari *et al.*, 2018 ▸, *L* = 1,10-phenanthroline, refcode: ZAMDOG; Parker & Brene­man, 1995 ▸; *L* = 2,2′-bi­pyridine, refcode: FERWEH; Tang *et al.*, 2017 ▸]. In Cu(NCS)I(pyridine)_4_·pyridine, the copper cations are octa­hedrally coordinated by one thio­cyanate anion, one iodide anion and four pyridine coligands (refcode: ESITOQ; Bowmaker *et al.*, 2011 ▸). In this compound, disorder is present with the iodide and thio­cyanate anions occupying the same crystallographic position. In a further copper compound, the copper cations are fivefold coordinated by one N and one S-bonding thio­cyanate anion, one chloride anion and two N atoms of the coligand (QETTER; Hu *et al.*, 2018 ▸). Two Cu^II^ cations are linked by pairs of μ-1,3-bridging thio­cyanate anions into dinuclear complexes. In di­aqua-bis­{μ-*N*′,*N*′′-[(pyridine-2,6-di­yl)bis­(eth-1-yl-1-yl­idene)]bis­(pyridine-4-car­bohydrazide)}bis­(iso­thio­cyanato)­tetra­chloro­trimanganese(II), one of the crystallographically independent manganese cations is octa­hedrally coordinated by two thio­cyanate anions, two chloride anions and two of the coligands (EWEVEK; Croitor *et al.*, 2021 ▸). One discrete complex with additional hydrate mol­ecules with the composition Mn(NCS)Cl(H_2_O)*L*·(H_2_O) is also reported in which the manganese cation is octa­hedrally coordinated by one thio­cyanate anion, one chloride anion and one tridentate coligand (*L* = 2,3,5,6-tetra­kis­(pyridin-2-yl)pyrazine, refcode: ZEYWUX; Machura *et al.*, 2013 ▸). Two discrete complexes of the composition Zn(NCS)Cl_2_ *L* exist in which the zinc cations are tetra­hedrally coordinated by two halide anions and one organic ligand (refcode: QINJEF; Kwiatek *et al.*, 2019 ▸). The fourth coord­ination site is mixed occupied by chloride and thio­cyanate anions in a 0.67:0.33 ratio. With a slightly modified ligand, a further compound is found that is isotypic to the former and in which the fourth position is exclusively coordinated by only thio­cyanate anions (refcode: QINJUV; Kwiatek *et al.*, 2019 ▸). With zinc, a further compound is known with composition Zn(NCS)Cl_2_(H_2_O)(phenanthroline) in which the zinc cation is octa­hedrally coordinated by one thio­cyanate anion, two chloride anions, one water ligand and one bidentate phenanthroline coligand (refcode: CUSVUI; Ma *et al.*, 2010 ▸). Finally, an additional compound with cadmium is known in which one of the two crystallographically independent cadmium cations is octa­hedrally coordinated by one thio­cyanate anion, two chloride anions and one bidentate {μ-2,2′,2′′-[1-(pyridin-2-ylmeth­yl)imidazolidine-2,4,5-tri­yl]tri­pyridine} coligand (refcode: DOWCUP; Ou *et al.*, 2014 ▸). The Cd^II^ cations are linked by μ-1,1-bridging chloride anions into chains.

## 5. Synthesis and crystallization

**Synthesis**

FeCl_2_·4H_2_O and KSCN were purchased from Sigma Aldrich and 3-cyano­pyridine was purchased from Alfa Aesar.

A microcrystalline powder was obtained by the reaction of 0.25 mmol of FeCl_2_·4H_2_O (49.7 mg), 0.25 mmol of KSCN (24.3 mmol) and 2 mmol of 3-cyano­pyridine (208.2 mg) in ethanol. The mixture was stirred for 1 d at room temperature, filtered off and washed with water. Crystals suitable for single-crystal X-ray diffraction were obtained using 0.25 mmol of FeCl_2_·4H_2_O (49.7 mg), 0.5 mmol of KSCN (48.6 mmol) and 2 mmol of 3-cyano­pyridine (208.2 mg) in ethanol under hydro­thermal conditions (403 K for 1 d).

**Figure 4:**
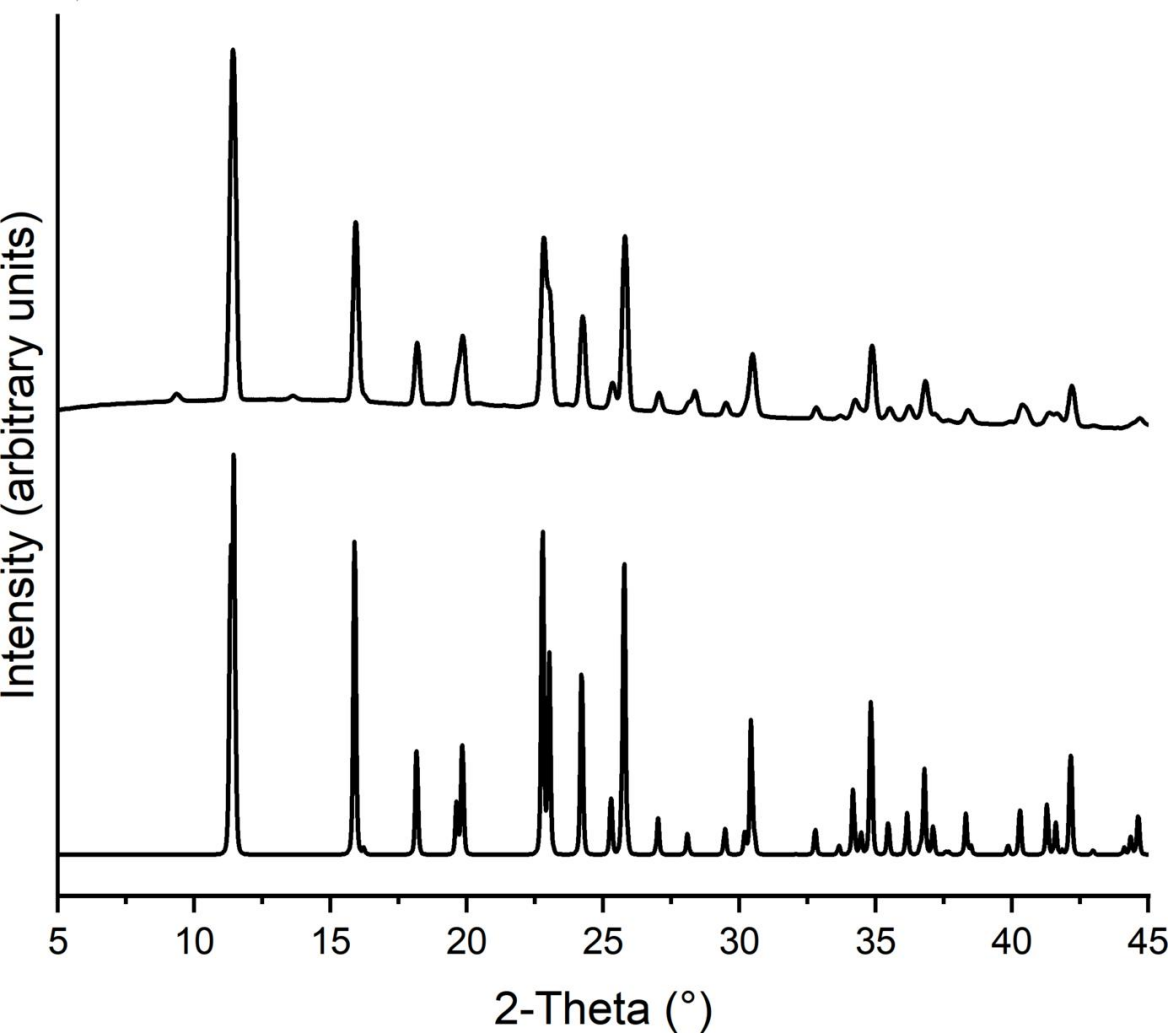
Experimental (top) and calculated (bottom) PXRD patterns of the title compound. Please note that the powder pattern was calculated using data from a structure determination performed at room temperature.

**Figure 5:**
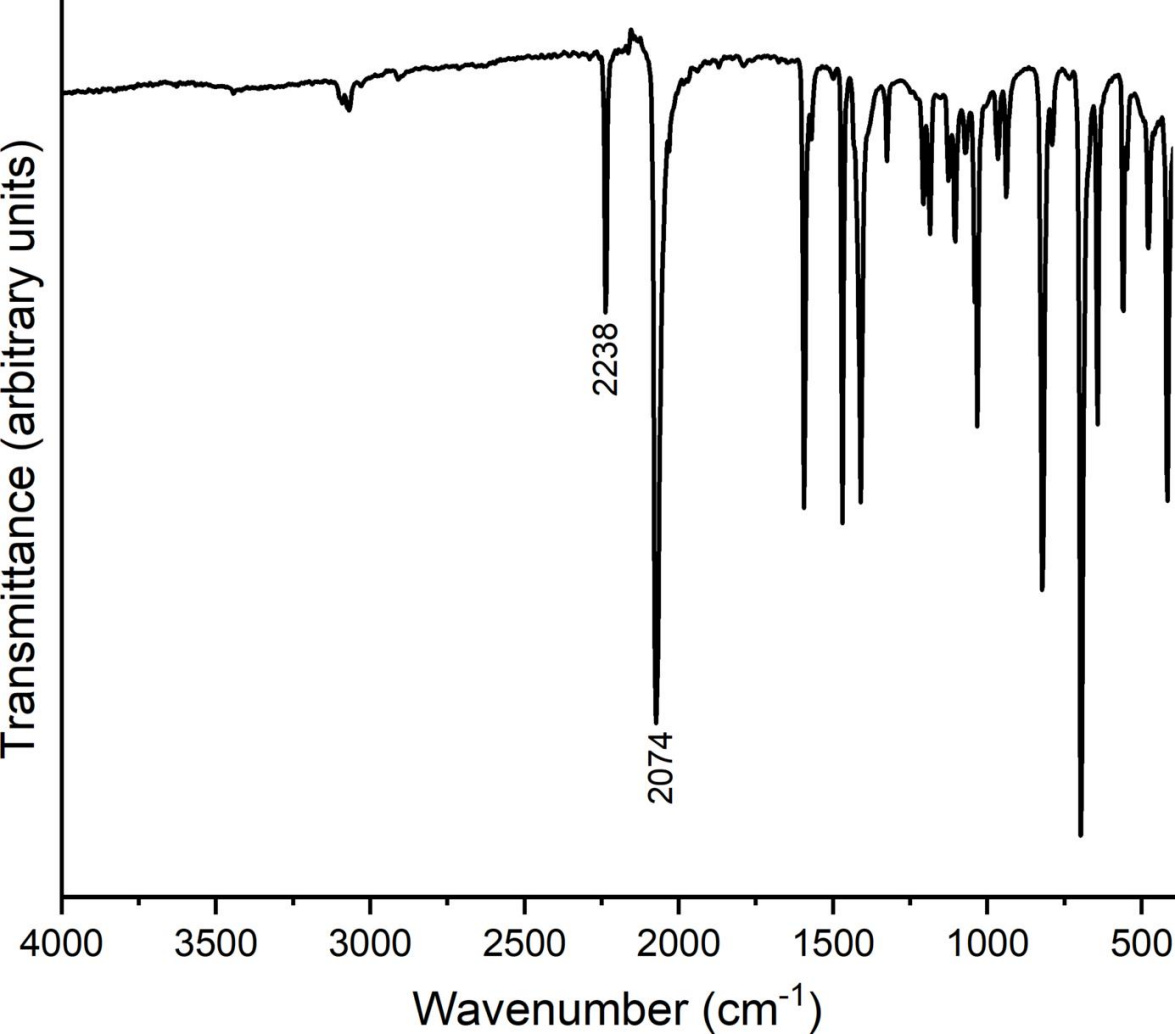
IR spectrum of the title compound. The CN stretching vibrations of the thio­cyanate anion and the cyano group of the 3-cyano­pyridine coligand are given.

Concerning the synthesis of the title complex, it is noted that in the beginning of our synthetic work, this compound was accidentally obtained by the reaction of one equivalent FeCl_2_·4H_2_O with two equivalents of KSCN. Comparison of the experimental powder pattern of this batch with that calculated from single-crystal data measured at room temperature shows that the title compound was obtained as the major phase, together with some amount of an unknown crystalline product (Fig. S1). Later on, the ratio between FeCl_2_·4H_2_O and KSN was reduced to 1:1, leading to title complex as a nearly pure phase (Fig. 4 ▸). However, there are a few additional reflections of low intensity that correspond to a small contamination of an unknown phase, which is different from the byproduct obtained by the reaction with a 1:2 ratio (Fig. 4 ▸). In the IR spectrum of the title compound, the CN stretching vibration of the thio­cyanate anions is observed at 2074 cm^−1^, which is in agreement with the presence of only terminally bonded thio­cyanate anions (Bailey *et al.*, 1971 ▸; Fig. 5 ▸). Moreover, the band at higher wavenumbers corresponds to the CN stretching vibration of the cyano group, for which a value of 2238 cm^−1^ is observed (Smith, 2019 ▸). This shows that the cyano group is not involved in the metal coordination (Reedijk & Groeneveld, 1967 ▸).

**Experimental details**

The data collection for single-crystal structure analysis and powder X-ray diffraction was performed using an XtaLAB Synergy, Dualflex, HyPix diffractometer from Rigaku with Cu *K*α radiation.

The IR spectrum was measured using an ATI Mattson Genesis Series FTIR Spectrometer, control software *WINFIRST*, from ATI Mattson.

Thermogravimetry and differential thermoanalysis (TG–DTA) measurements were performed in a dynamic air atmosphere in Al_2_O_3_ crucibles using a STA-PT 1000 thermobalance from Linseis. The instrument was calibrated using standard reference materials.

## 6. Thermogravimetry and differential thermoanalysis

The thermal properties of the title compound were investigated by thermogravimetry and differential thermoanalysis (TG–DTA). Upon heating, two mass losses were observed that, according to the DTG curve, are poorly resolved and that are accompanied with two endothermic events in the DTA curve (Fig. S2). The experimental mass loss in the first step is in rough agreement with that calculated for the removal of two 3-cyano­pyridine ligands of 36.8%, whereas the value for the second mass loss is lower. This indicates that a compound with the composition Fe(NCS)(Cl)(3-cyano­pyridine)_2_ has formed after the first mass loss. Powder X-ray diffraction reveals that in the residue obtained after the first mass loss, no reflections of the pristine compound are present and that a phase of poor crystallinity has formed (Fig. S3). IR measurements of this residue show that the CN stretching vibration of the thio­cyanate anion is shifted to 2025 cm^−1^, whereas the CN stretching vibration of the cyano group remains constant. This strongly indicates that the μ-1,3-bridging thio­cyanate anions are present and that the cyano group is still not involved in the metal coordination. In most cases, the structures of compounds with such a stoichiometry consist of chains in which the metal centers are octa­hedrally coordinated and linked by pairs of μ-1,3-bridging thio­cyanate anions into chains (Jochim *et al.*, 2018 ▸; Wöhlert *et al.*, 2013 ▸; Mautner *et al.*, 2018 ▸). Alternatively, a layered structure has formed in which the metal cations are octa­hedrally coordinated and linked by single bridging anionic ligands into layers (Werner *et al.*, 2015*b* ▸; Jochim *et al.*, 2020*a* ▸,*b* ▸) or two metal cations are linked by pairs of thio­cyanate anions into dinuclear units that are further connected into layers by single μ-1,3-bridging anionic ligands (Suckert *et al.*, 2016 ▸). Other topologies of thio­cyanate networks are very rare.

## 7. Refinement

Crystal data, data collection and structure refinement details are summarized in Table 3 ▸. C-bound H atoms were positioned with idealized geometry (C—H = 0.95 Å) and refined isotropically with *U*_iso_(H) = 1.2*U*_eq_(C) using a riding model. The absolute structure was determined and is in agreement with the selected setting.

## Supplementary Material

Crystal structure: contains datablock(s) I. DOI: 10.1107/S205698902300988X/nx2002sup1.cif

Structure factors: contains datablock(s) I. DOI: 10.1107/S205698902300988X/nx2002Isup2.hkl

Experimental (top) powder pattern of the product obtained by the synthesis of the title compound using FeCL2 . 4 H2O and KNCS in ratio 1:2 and calculated pattern for the title complex (bottom). DOI: 10.1107/S205698902300988X/nx2002sup3.png

DTG, TG and DTA curve for the title complex. DOI: 10.1107/S205698902300988X/nx2002sup4.png

Experimental (top) powder pattern of the intermediate compound obtained after the first mass loss in a TG measurement of the title complex together with the pattern of the title complex calculated using data measured at room temperature (bottom). DOI: 10.1107/S205698902300988X/nx2002sup5.png

IR spectra of the intermediate compound obtained after the first mass loss in a TG measurement of the title compound. DOI: 10.1107/S205698902300988X/nx2002sup6.png

CCDC reference: 2307914

Additional supporting information:  crystallographic information; 3D view; checkCIF report

## Figures and Tables

**Table 1:** Selected geometric parameters (Å, °)

Fe1—N1	2.099 (4)	Fe1—N11	2.2480 (18)
Fe1—Cl1	2.3716 (12)		
			
N1—Fe1—Cl1	180.0	N11^i^—Fe1—N11^ii^	89.546 (11)
N1—Fe1—N11	84.89 (6)	N11^i^—Fe1—N11	169.79 (12)
N11—Fe1—Cl1	95.11 (6)	N11—Fe1—N11^iii^	89.545 (11)

Symmetry codes: (i) 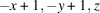 ; (ii) 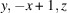 ; (iii) 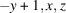 .

**Table 2:** Hydrogen-bond geometry (Å, °)

*D*—H⋯*A*	*D*—H	H⋯*A*	*D*⋯*A*	*D*—H⋯*A*
C14—H14⋯N12^iv^	0.95	2.68	3.304 (3)	124
C15—H15⋯N12^iv^	0.95	2.67	3.313 (3)	126

Symmetry code: (iv) 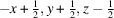 .

**Table 3:** Experimental details

Crystal data	
Chemical formula	[FeCl(NCS)(C_6_H_4_N_2_)_4_]
*M*_r_	565.83
Crystal system, space group	Tetragonal, *P*4*n* *c*
Temperature (K)	100
*a*, *c* (Å)	10.79412 (6), 11.15065 (11)
*V* (Å^3^)	1299.20 (2)
*Z*	2
Radiation type	Cu *K*α
μ (mm^−1^)	6.62
Crystal size (mm)	0.18 × 0.08 × 0.06

Data collection	
Diffractometer	XtaLAB Synergy, Dualflex, HyPix
Absorption correction	Multi-scan (*CrysAlis PRO*; Rigaku OD, 2023 ▸)
*T*_min_, *T*_max_	0.686, 1.000
No. of measured, independent and observed [*I* > 2σ(*I*)] reflections	11711, 1411, 1406
*R*_int_	0.021
(sin θ/λ)_max_ (Å^−1^)	0.638

Refinement	
*R*[*F* ^2^ > 2σ(*F* ^2^)], *wR*(*F* ^2^), *S*	0.023, 0.065, 1.14
No. of reflections	1411
No. of parameters	88
No. of restraints	1
H-atom treatment	H-atom parameters constrained
Δρ_max_, Δρ_min_ (e Å^−3^)	0.34, −0.35
Absolute structure	Flack *x* determined using 652 quotients [(*I* ^+^)−(*I* ^−^)]/[(*I* ^+^)+(*I* ^−^)] (Parsons *et al.*, 2013 ▸)
Absolute structure parameter	−0.0059 (19)

Computer programs: *CrysAlis PRO* (Rigaku OD, 2023 ▸), *SHELXT2014/5* (Sheldrick, 2015*a* ▸), *SHELXL2016/6* (Sheldrick, 2015*b* ▸), *DIAMOND* (Brandenburg & Putz, 1999 ▸) and *publCIF* (Westrip, 2010 ▸).

## supplementary crystallographic information

### Crystal data

**Table:**

[FeCl(NCS)(C_6_H_4_N_2_)_4_]	*D*_x_ = 1.446 Mg m^−^^3^
*M**_r_* = 565.83	Cu *K*α radiation, λ = 1.54184 Å
Tetragonal, *P*4*n**c*	Cell parameters from 9200 reflections
*a* = 10.79412 (6) Å	θ = 5.7–79.7°
*c* = 11.15065 (11) Å	µ = 6.62 mm^−^^1^
*V* = 1299.20 (2) Å^3^	*T* = 100 K
*Z* = 2	Block, yellow
*F*(000) = 576	0.18 × 0.08 × 0.06 mm

### Data collection

**Table:**

XtaLAB Synergy, Dualflex, HyPix diffractometer	1411 independent reflections
Radiation source: micro-focus sealed X-ray tube, PhotonJet (Cu) X-ray Source	1406 reflections with *I* > 2σ(*I*)
Mirror monochromator	*R*_int_ = 0.021
Detector resolution: 10.0000 pixels mm^-1^	θ_max_ = 79.9°, θ_min_ = 5.7°
ω scans	*h* = −13→12
Absorption correction: multi-scan (CrysAlisPro; Rigaku OD, 2023)	*k* = −13→13
*T*_min_ = 0.686, *T*_max_ = 1.000	*l* = −14→13
11711 measured reflections	

### Refinement

**Table:**

Refinement on *F*^2^	Hydrogen site location: inferred from neighbouring sites
Least-squares matrix: full	H-atom parameters constrained
*R*[*F*^2^ > 2σ(*F*^2^)] = 0.023	*w* = 1/[σ^2^(*F*_o_^2^) + (0.0384*P*)^2^ + 0.3704*P*] where *P* = (*F*_o_^2^ + 2*F*_c_^2^)/3
*wR*(*F*^2^) = 0.065	(Δ/σ)_max_ < 0.001
*S* = 1.14	Δρ_max_ = 0.34 e Å^−^^3^
1411 reflections	Δρ_min_ = −0.34 e Å^−^^3^
88 parameters	Absolute structure: Flack *x* determined using 652 quotients [(*I*^+^)-(*I*^-^)]/[(*I*^+^)+(*I*^-^)] (Parsons *et al.*, 2013)
1 restraint	Absolute structure parameter: −0.0059 (19)
Primary atom site location: dual	

### Special details

**Table:**

Geometry. All esds (except the esd in the dihedral angle between two l.s. planes) are estimated using the full covariance matrix. The cell esds are taken into account individually in the estimation of esds in distances, angles and torsion angles; correlations between esds in cell parameters are only used when they are defined by crystal symmetry. An approximate (isotropic) treatment of cell esds is used for estimating esds involving l.s. planes.

### Fractional atomic coordinates and isotropic or equivalent isotropic displacement parameters (Å^2^)

**Table:**

	*x*	*y*	*z*	*U*_iso_*/*U*_eq_	
Fe1	0.500000	0.500000	0.61306 (6)	0.01647 (19)	
N1	0.500000	0.500000	0.4248 (4)	0.0230 (11)	
C1	0.500000	0.500000	0.3226 (6)	0.0211 (10)	
S1	0.500000	0.500000	0.17570 (13)	0.0298 (3)	
Cl1	0.500000	0.500000	0.82574 (10)	0.0218 (3)	
N11	0.37363 (17)	0.33550 (16)	0.59511 (18)	0.0205 (4)	
C11	0.38831 (19)	0.2322 (2)	0.6601 (2)	0.0215 (4)	
H11	0.442330	0.233778	0.727352	0.026*	
C12	0.3268 (2)	0.12224 (19)	0.6321 (2)	0.0231 (4)	
C13	0.2477 (2)	0.1182 (2)	0.5330 (2)	0.0247 (5)	
H13	0.206877	0.043560	0.511051	0.030*	
C14	0.2307 (2)	0.2254 (2)	0.4677 (3)	0.0235 (5)	
H14	0.176720	0.226567	0.400458	0.028*	
C15	0.2940 (2)	0.3315 (2)	0.5023 (2)	0.0216 (4)	
H15	0.280313	0.405460	0.457985	0.026*	
C16	0.3447 (2)	0.0135 (2)	0.7051 (3)	0.0278 (5)	
N12	0.3583 (2)	−0.0743 (2)	0.7610 (2)	0.0362 (5)	

### Atomic displacement parameters (Å^2^)

**Table:**

	*U*^11^	*U*^22^	*U*^33^	*U*^12^	*U*^13^	*U*^23^
Fe1	0.0139 (2)	0.0139 (2)	0.0216 (4)	0.000	0.000	0.000
N1	0.0229 (14)	0.0229 (14)	0.023 (2)	0.000	0.000	0.000
C1	0.0117 (14)	0.0117 (14)	0.040 (3)	0.000	0.000	0.000
S1	0.0322 (5)	0.0322 (5)	0.0251 (6)	0.000	0.000	0.000
Cl1	0.0210 (4)	0.0210 (4)	0.0234 (6)	0.000	0.000	0.000
N11	0.0171 (8)	0.0169 (8)	0.0275 (9)	−0.0010 (6)	−0.0007 (8)	0.0010 (7)
C11	0.0167 (9)	0.0202 (10)	0.0276 (10)	0.0000 (8)	0.0006 (8)	0.0007 (9)
C12	0.0205 (10)	0.0177 (10)	0.0311 (11)	−0.0003 (8)	0.0030 (8)	0.0027 (9)
C13	0.0220 (10)	0.0205 (10)	0.0315 (12)	−0.0043 (8)	0.0017 (9)	−0.0028 (9)
C14	0.0186 (10)	0.0235 (11)	0.0283 (10)	−0.0038 (9)	−0.0019 (10)	−0.0016 (10)
C15	0.0174 (9)	0.0198 (10)	0.0276 (10)	−0.0011 (7)	−0.0037 (9)	0.0015 (9)
C16	0.0223 (10)	0.0219 (11)	0.0391 (14)	−0.0026 (8)	0.0021 (11)	0.0037 (10)
N12	0.0326 (10)	0.0273 (11)	0.0488 (12)	−0.0001 (8)	0.0037 (10)	0.0107 (10)

### Geometric parameters (Å, º)

**Table:**

Fe1—N1	2.099 (4)	C11—H11	0.9500
Fe1—Cl1	2.3716 (12)	C11—C12	1.395 (3)
Fe1—N11^i^	2.2480 (18)	C12—C13	1.397 (3)
Fe1—N11^ii^	2.2480 (18)	C12—C16	1.441 (3)
Fe1—N11	2.2480 (18)	C13—H13	0.9500
Fe1—N11^iii^	2.2480 (18)	C13—C14	1.379 (3)
N1—C1	1.140 (8)	C14—H14	0.9500
C1—S1	1.638 (7)	C14—C15	1.389 (3)
N11—C11	1.340 (3)	C15—H15	0.9500
N11—C15	1.346 (3)	C16—N12	1.144 (3)
			
N1—Fe1—Cl1	180.0	C11—N11—C15	117.71 (19)
N1—Fe1—N11^i^	84.89 (6)	C15—N11—Fe1	118.75 (14)
N1—Fe1—N11^ii^	84.89 (6)	N11—C11—H11	119.0
N1—Fe1—N11	84.89 (6)	N11—C11—C12	122.1 (2)
N1—Fe1—N11^iii^	84.89 (6)	C12—C11—H11	119.0
N11^iii^—Fe1—Cl1	95.11 (6)	C11—C12—C13	119.6 (2)
N11^i^—Fe1—Cl1	95.11 (6)	C11—C12—C16	120.1 (2)
N11^ii^—Fe1—Cl1	95.11 (6)	C13—C12—C16	120.2 (2)
N11—Fe1—Cl1	95.11 (6)	C12—C13—H13	120.9
N11^ii^—Fe1—N11^i^	89.546 (11)	C14—C13—C12	118.2 (2)
N11—Fe1—N11^i^	89.547 (11)	C14—C13—H13	120.9
N11^ii^—Fe1—N11^iii^	89.546 (11)	C13—C14—H14	120.6
N11^i^—Fe1—N11^iii^	169.78 (12)	C13—C14—C15	118.7 (2)
N11^ii^—Fe1—N11	169.79 (12)	C15—C14—H14	120.6
N11—Fe1—N11^iii^	89.545 (11)	N11—C15—C14	123.6 (2)
C1—N1—Fe1	180.0	N11—C15—H15	118.2
N1—C1—S1	180.0	C14—C15—H15	118.2
C11—N11—Fe1	122.53 (15)	N12—C16—C12	178.5 (3)

Symmetry codes: (i) *y*, −*x*+1, *z*; (ii) −*x*+1, −*y*+1, *z*; (iii) −*y*+1, *x*, *z*.

### Hydrogen-bond geometry (Å, º)

**Table:**

*D*—H···*A*	*D*—H	H···*A*	*D*···*A*	*D*—H···*A*
C14—H14···N12^iv^	0.95	2.68	3.304 (3)	124
C15—H15···N12^iv^	0.95	2.67	3.313 (3)	126

Symmetry code: (iv) −*x*+1/2, *y*+1/2, *z*−1/2.
