# Supplementary figures and images for: Synthesis, crystal structure and properties of chlorido­tetra­kis­(pyridine-3-carbo­nitrile)­thio­cyanato­iron(II)
Source: Acta Crystallogr E Crystallogr Commun. 2023 Nov 21;79(Pt 12):1173–8. doi: 10.1107/S205698902300988X (PMC10833408; doi:10.1107/S205698902300988X)

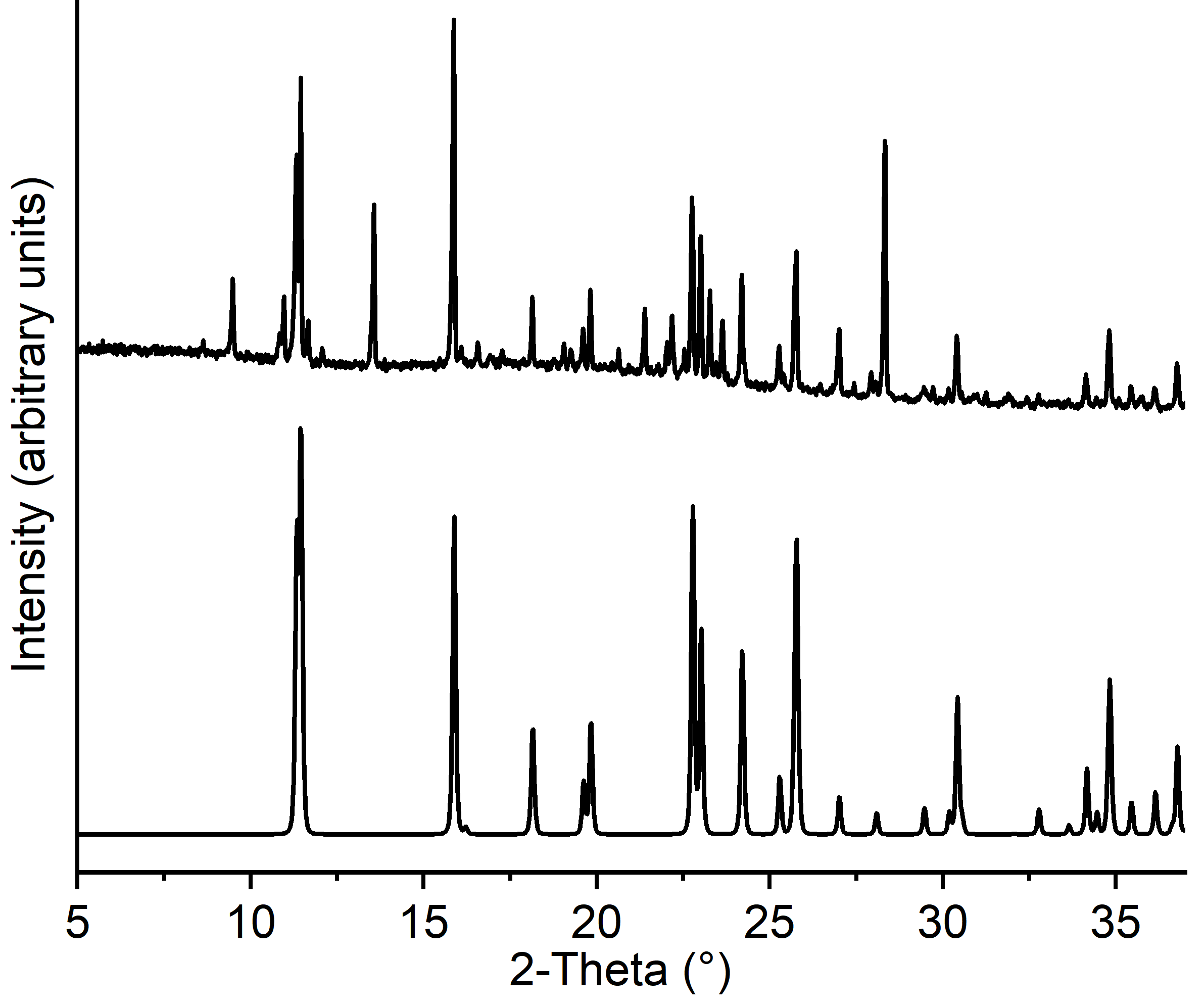

Supplement: Supplementary file 3 [file e-79-01173-sup3.png]

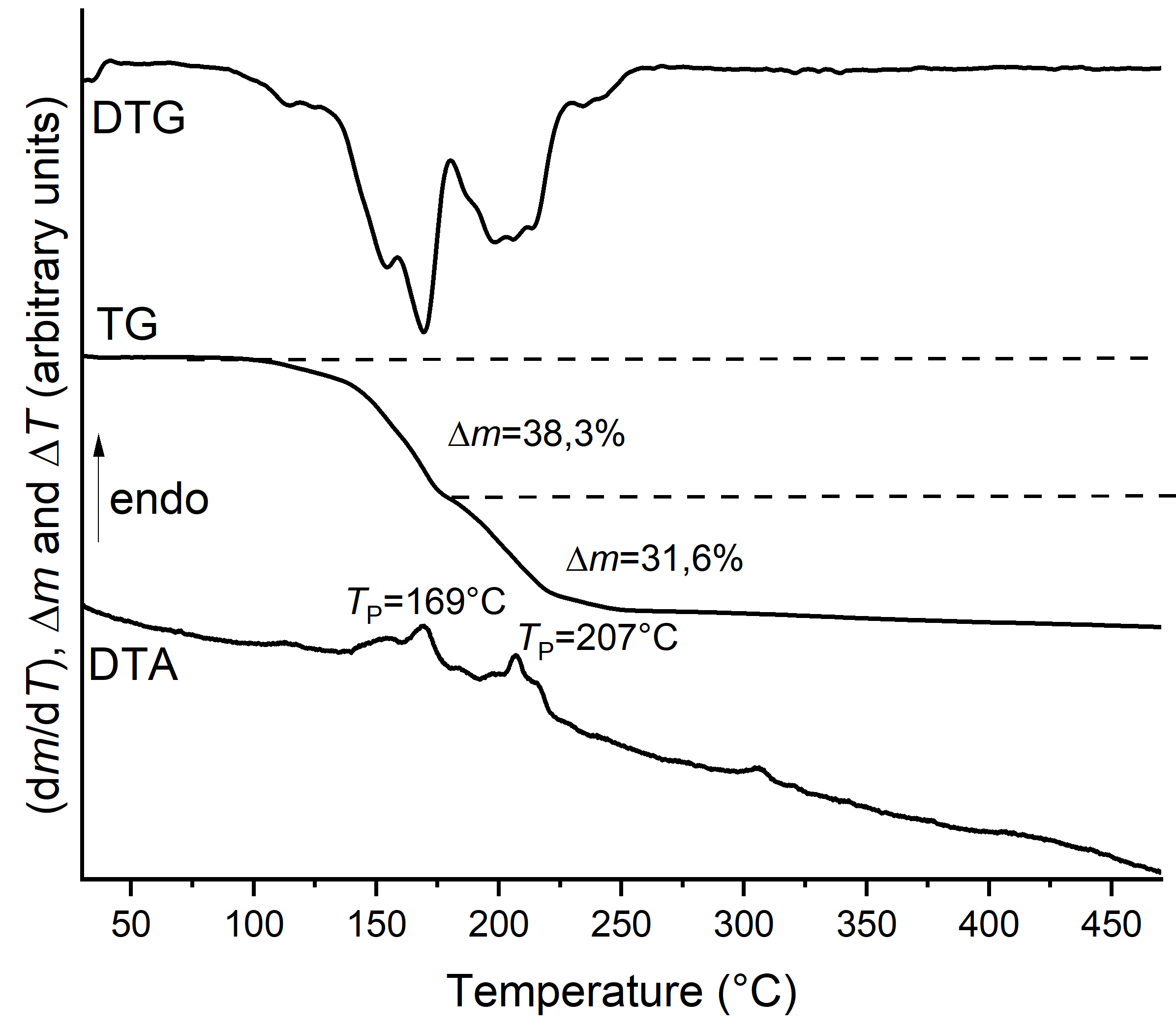

Supplement: Supplementary file 4 [file e-79-01173-sup4.png]

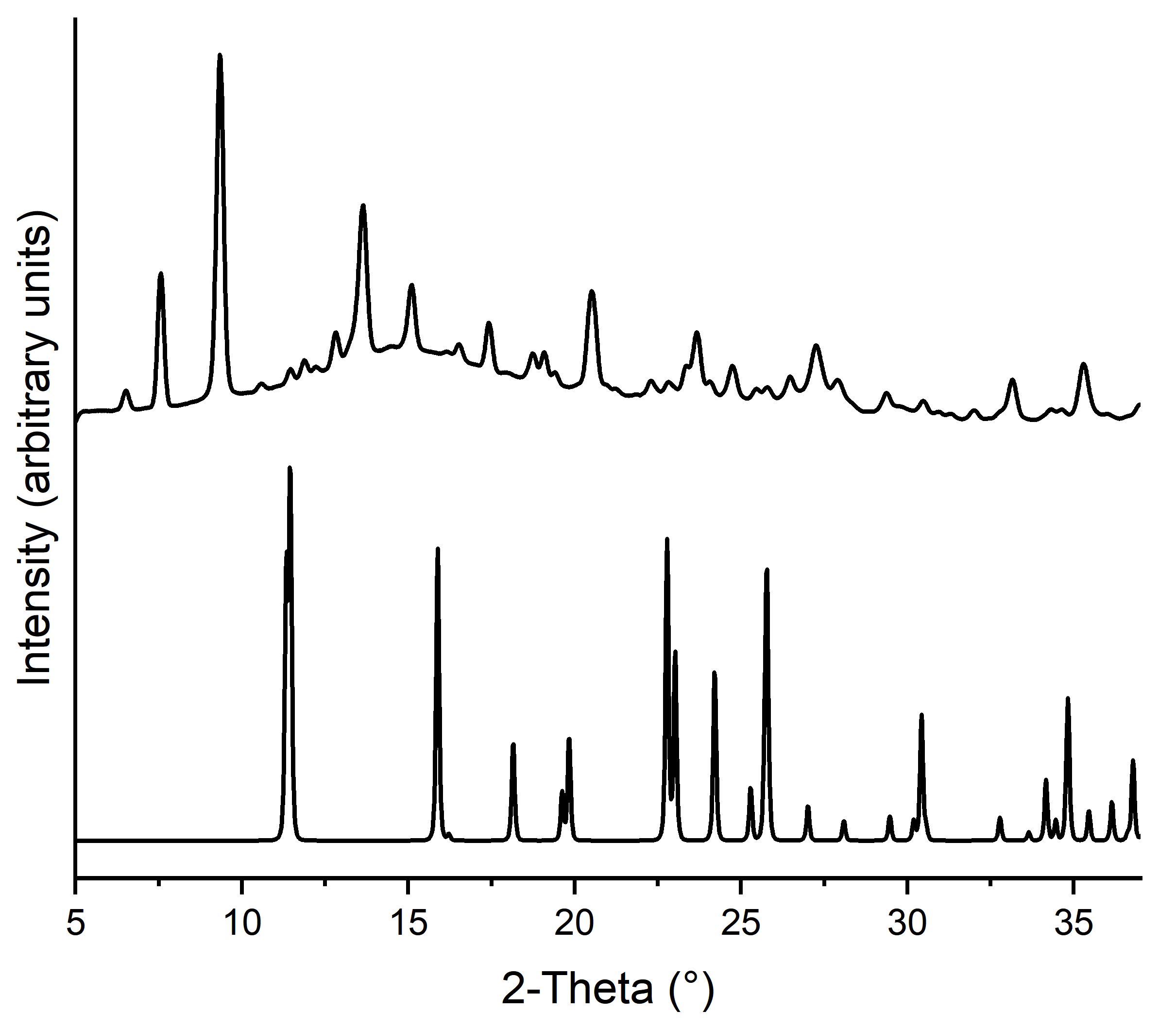

Supplement: Supplementary file 5 [file e-79-01173-sup5.png]

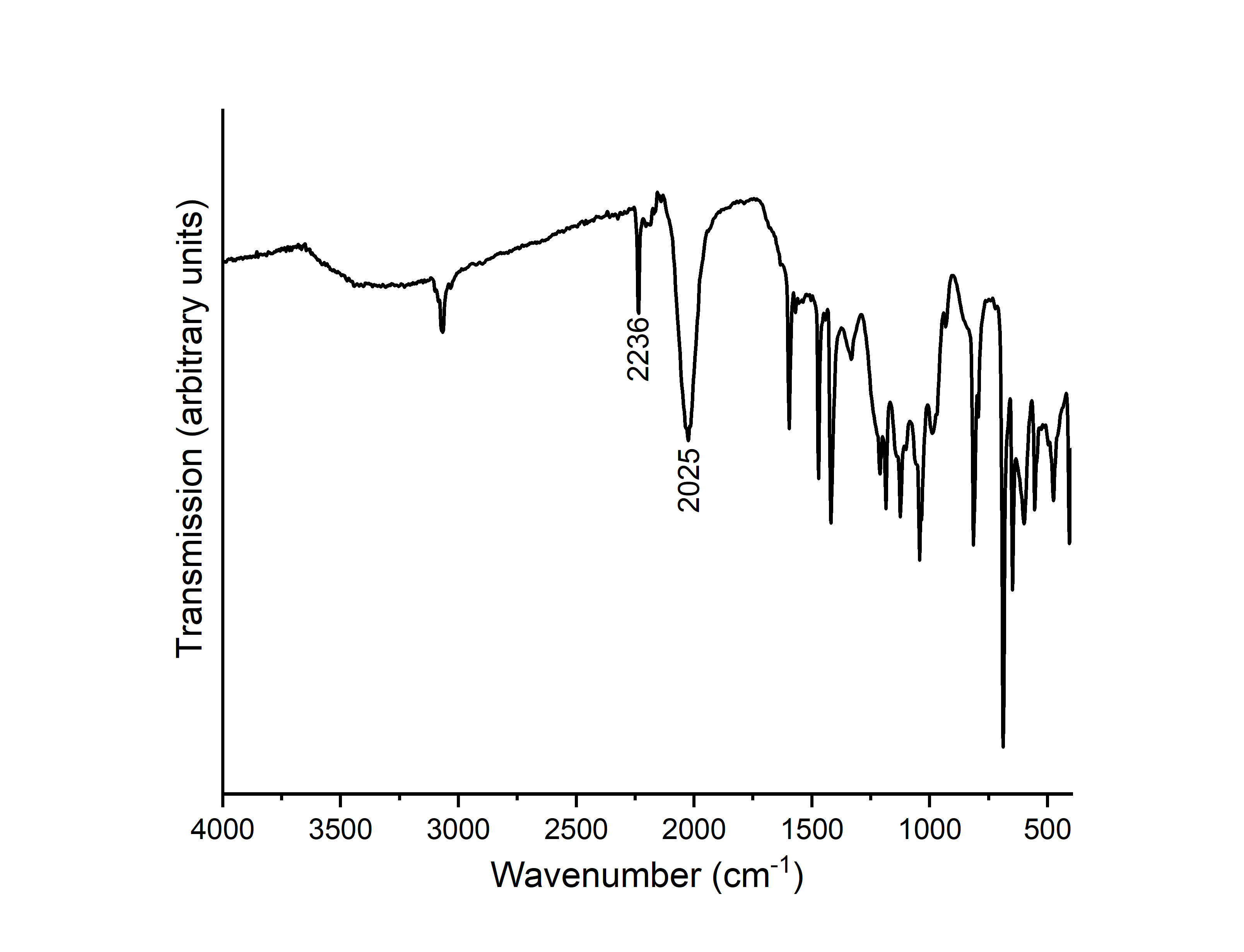

Supplement: Supplementary file 6 [file e-79-01173-sup6.png]
